# Shifting partisan public opinion towards Community Choice Aggregation through outreach and awareness

**DOI:** 10.1371/journal.pone.0292136

**Published:** 2023-10-03

**Authors:** Victor Y. Wu, Richard Howarth

**Affiliations:** 1 Stanford Law School, Stanford University, Stanford, California, United States of America; 2 Department of Environmental Studies, Dartmouth College, Hanover, New Hampshire, United States of America; Sichuan Agricultural University, CHINA

## Abstract

Community Choice Aggregation (CCA) is a rapidly expanding electricity supply model in the United States. By enabling local governments to obtain electricity for their residents, CCAs have the potential to increase the use of renewable energy while lowering costs. Recent studies have focused on how CCAs will impact renewable energy markets, since at least five more states are currently considering CCA-enabling legislation. However, little research has been done on partisan attitudes towards CCAs or how to shift public opinion to support the continued expansion of CCAs. We use a nationally representative survey experiment of 1,862 respondents to evaluate outreach and awareness campaigns by nonprofit and government organizations seeking to expand CCAs. We find that most Americans are currently unfamiliar with CCAs, but exposing them to educational outreach information increases their support for CCAs at the state, local, and personal levels. Furthermore, this information is equally effective at increasing support among both Democrats and Republicans, despite partisan polarization on renewable energy. However, this information did not significantly affect respondents’ price sensitivity with regard to CCAs. Our results suggest that outreach and awareness campaigns can be effective at increasing support for CCA among both Democrats and Republicans.

## Introduction

Most people living in the United States receive electricity from private investor-owned utilities, or IOUs [1]. However, ten states have enacted legislation that authorizes local governments to purchase electricity on behalf of their residents and businesses through Community Choice Aggregation programs, or CCAs [2]. By aggregating the purchasing power of residents, CCAs allow local governments to lower the cost of electricity while simultaneously increasing the use of renewable energy and giving residents more control over the composition of their energy mix (the mix of sources from which electricity is produced) [3, 4].

CCAs are expanding incredibly rapidly. In 2020, CCAs procured about 13 billion kWh of electricity for roughly 5 million customers [2]; in 2021, CCAs served over 11 million customers in California alone [5]. CCAs have “since been adopted by more than 1800 local governments that represent more than 36 million people in six states” [6]. CCAs have been authorized in ten states: California, Illinois, Maryland, Massachusetts, New Hampshire, New Jersey, New York, Ohio, Rhode Island, and Virginia [2]. As of 2023, at least five other states (Arizona, Colorado, Michigan, New Mexico, and Pennsylvania) are considering CCA-enabling legislation [7].

Despite their potential to reduce both electricity costs and greenhouse gas emissions [3, 4], CCAs have faced political opposition from some groups. Efforts to establish new CCAs have encountered resistance in the form of “lobbying, ballot measures, legislation and marketing campaigns” [8]. Partisan disagreements have already become entrenched for many areas of energy policy [9–11]; researchers should consider how to counteract increasing polarization around CCAs before attitudes towards CCAs also become irreversibly entangled with partisan politics. CCAs have expanded rapidly in Democratic-leaning states and communities, but their spread to Republican-leaning states and communities may face much greater political challenges, given partisan polarization on energy policy and renewables [12, 13].

Many IOUs have staunchly opposed the spread of CCAs. In 2010, the California Public Utilities Commission sent Pacific Gas and Electric Company (PG&E) a warning letter after finding that the IOU had been sending customers “misleading” mailers about CCAs and calling to convince customers not to affiliate with CCAs [14]. In 2017, Sempra Energy, the parent company of San Diego Gas and Electric (SDG&E), spent months lobbying elected officials against CCAs [15]. In 2022, the Public Service Company of Colorado (an IOU also known as “Xcel Energy”) filed comments to the Colorado Public Utilities Commission calling CCA a “wolf in sheep’s clothing” [16].

Although the rapid expansion of CCAs has spurred growing scholarly interest in the impacts of CCAs on the renewable energy market [4, 17–19], little research has been done on attitudes towards CCAs. No academic study thus far has attempted to directly measure public opinion regarding CCAs, despite the important role of public support in shaping policy [20, 21]. For example, Faruqui et al. [22] surveyed community and industry experts about CCAs in their own particular communities, while O’Shaughnessy et al. [3] interviewed “CCAs, CCA-focused organizations, and other stakeholders” and compiled survey data collected by CCAs on renewable energy sales. None of these data measure public opinion regarding CCAs. Bartling [23] attempted to measure public support for CCAs using referendum data from cities, towns and villages in Illinois. However, such data are, by definition, limited to the people who voted in referendums. Resulting selection bias may lead to overestimation of CCA support among the general public, as those who vote in a referendum on CCAs may be doing so precisely because they already hold uncommonly strong opinions about CCAs.

We attempt to fill this gap in the empirical literature by conducting a nationwide survey of 1,862 respondents. A nationwide survey helps us evaluate public support for CCA expansion to new states, which is important because at least five other states are currently considering CCA-enabling legislation [7]. A survey of only the 10 states which currently have such legislation would only allow us to evaluate support for CCA expansion within those 10 states. Additionally, a nationwide survey allows us to compare potential differences in attitudes between residents in states with and without CCA-enabling legislation. Hsu [6] writes that “formation of CCAs in multiple states proves that there is a widespread desire for local control of energy policy, utilities, and infrastructure,” but is this desire confined to the ten states with CCA-enabling legislation? Or does this desire extend to other states that have not yet enacted CCA-enabling legislation? Since nonprofits such as LEAN Energy US are “dedicated to the accelerated expansion and competitive success of clean energy CCA *nationwide*” (emphasis added), we wanted to test whether the educational messaging strategy of these nonprofits works even in states lacking CCA-enabling legislation [24]. Finally, an additional benefit of a nationwide survey is that it provides some measure of public support for a federal authorization of CCA in every state [25].

We find that most respondents are unfamiliar with CCAs, but giving people educational outreach information about CCAs and energy mixes increases their support for CCAs at the state, local, and personal levels. The treatment was equally effective for Republicans and Democrats; one group was not significantly more influenced by the treatment than another group, despite partisan polarization on renewable energy and the environment. This suggests that policymakers and nonprofits seeking to expand CCA adoption (either to reduce electricity prices or mitigate climate change) should increase education and public awareness about CCAs. Though we do not find treatment effects on price sensitivity, outreach campaigns may not need to reduce price sensitivity as CCAs already promote the cost-saving feature of their default energy mix.

### Community Choice Aggregations (CCAs)

The U.S. Environmental Protection Agency (EPA) defines CCAs as programs that “allow local governments to procure power on behalf of their residents, businesses, and municipal accounts from an alternative supplier while still receiving transmission and distribution service from their existing utility provider” [2]. CCA, also known as “Municipal Aggregation,” “Government Energy Aggregation,” or “Community Choice Energy,” was first created in Massachusetts by the 1997 Electricity Restructuring Act; in 1998, the Cape Light Compact became the first CCA in the United States [6]. Since the existing utility provider is still responsible for transmission, distribution, and other customer services, CCAs are often described as a “hybrid utility model” [19].

CCAs determine the mix of sources used to supply electricity, also known as an “electricity mix” or “electricity portfolio.” O’Shaughnessy et al. [3] describe how CCAs are required to comply with state renewable portfolio standards (RPS)—“a state-level policy mandating that load-serving entities procure a specified amount of their electricity portfolios from renewable energy generators.” However, CCAs often choose to go above the minimum percentage of renewables set by RPS. Indeed, a major attraction of CCAs is precisely their efforts to increase the percentage of renewables in both the CCA’s default energy mix as well as their premium energy mix or mixes.

Crucial to the success of CCAs is their opt-out structure. O’Shaughnessy et al. [3] note that “the opt-out structure is the key distinguishing feature between CCAs and other voluntary renewable energy programs”; since decision makers tend to follow the path of least resistance, empirical data from behavioral economics has found a “default bias” whereby people are biased toward the “option that will occur if the decision maker takes no action.” O’Shaughnessy et al. thus argue that “high participation rates in CCAs may reflect the default bias in action.” Furthermore, the opt-out structure enabled CCAs to gain support from more legislators in Massachusetts during 1997 [6]. Since legislators today are likely still reluctant to force people to participate in CCAs, the existence of an “opt-out” option may continue to improve the odds of CCA-enabling legislation passing at the state level and CCAs being implemented at the local level.

Most people enrolled in a CCA are assigned the CCA’s default energy mix, which is usually less expensive *and* includes a higher percentage of renewables than the IOU’s default energy mix [3]. However, one can choose to “opt-up” to energy mixes with a higher percentage of renewables that are often more expensive.

## Methods

### Sample

We collected data from respondents surveyed by Lucid Theorem from April 19–May 1, 2022 using a nationally representative online sample balanced based on age, gender, ethnicity, and region. We excluded respondents who did not consent to the survey, were under 18, did not reside in the United States, or did not pass both of our attention checks. The final sample consisted of 1,862 respondents.

We also conducted a pretest of 350 respondents from March 11–March 19, 2022 using a nationally representative online sample from Lucid Theorem, in order to test the experimental design, gauge respondent reactions to the treatment language, and evaluate responses to the manipulation checks. We do not include the data from the pretest in our sample. For both the pretest and the main survey, we obtained written consent for all respondents. The study was approved by the Committee for the Protection of Human Subjects at Dartmouth College (STUDY00032455).

We sorted the sample into Democrats, Republicans, and pure independents for analysis. (Following conventional practices, we treat partisan leaners as partisans, based on prior research finding that partisan leaners behave the same as partisans [26–29].) We choose to analyze attitudes towards CCAs in terms of partisanship because of increasing partisan polarization on the environment and energy policy [12, 13]. In the United States, political orientation is strongly associated with interest in and use of renewable energy [30, 31].

### Measuring support for CCAs

Individual participation in a CCA can generally be thought of as occurring in three stages. First, one’s state passes CCA-enabling legislation, which authorizes local governments to implement CCAs. Second, one’s local government implements a CCA by holding a vote or passing a public referendum [32]. Third, one “chooses” to participate in the CCA. Most CCAs are “opt-out,” meaning that once a local government implements a CCA, all residents are automatically enrolled in the CCA after a certain period of time [2]. Thus, choosing to participate in a CCA generally means “choosing” to *not opt out* of the CCA.

We measure support for CCAs at each of these three stages. We wanted to answer the following questions: 1) Do respondents support their state passing CCA-enabling legislation? 2) Do respondents support their local government implementing a CCA? 3) Would respondents personally choose to participate in a CCA? Thus, to measure support for CCAs at the state, local, and personal levels, respondents were asked to rate their agreement with the three following statements:

1. “My state should have Community Choice Aggregation (CCA) legislation, which authorizes local governments to decide whether they want to implement CCAs.”
2. “My local government should implement a Community Choice Aggregation (CCA), which automatically enrolls each local resident in the CCA unless he or she decides to opt out.”
3. “Assuming that the price of my energy would be roughly the same or slightly lower, I would participate in a Community Choice Aggregation (CCA).”

For the third statement, we asked respondents to assume their energy prices would be “roughly the same or slightly lower” because CCAs’ default energy mixes are indeed usually less expensive than the IOUs’ default energy mixes, and this benefit is an important aspect of CCA messaging [3, 4]. Across all three measures, variables were coded so that higher values implied more support for CCAs. We use factor analysis to create a composite scale of these three measures, since all three loaded together based on the factor analysis results; we also calculate the mean of these three measures. We analyze and report results for the composite scale and the mean as well as for each of the three measures separately.

### Measuring price sensitivity with regards to CCAs

We also evaluate respondents’ price sensitivity regarding CCA participation. To measure price sensitivity, respondents were asked to rate their agreement with the three following statements (key words were bolded for respondents):

1. “I would participate in a Community Choice Aggregation (CCA) **even if** my electricity bill would go up.”
2. “I would participate in a Community Choice Aggregation (CCA) **only if** my electricity bill would go down.”
3. “I would participate in a Community Choice Aggregation (CCA) **regardless** of how my electricity bill might change.”

These three measures will be referred to as “Even if up,” “Only if down,” and “Regardless change,” respectively. Although there are many ways to measure price sensitivity, other studies have also used Likert scales [33, 34]. As a fourth measure of price sensitivity, respondents were asked to select the potential benefit of CCAs that they found most appealing: “More renewable sources of energy,” “Lower electricity bill,” or “More local control over energy.” This measure will be referred to as “Monetary benefit.” For the “Monetary benefit” measure, “Lower electricity bill” was coded as 1; the other two options were coded as 0. This measure thus represents respondents’ preference for the monetary benefits of CCAs.

Across all four measures, variables were coded so that higher values implied higher price sensitivity with regard to CCAs. We use factor analysis to create a composite scale of the three measures that loaded together based on the factor analysis results (“Even if up,” “Regardless change,” and “Monetary benefit”); we also calculate the mean of the two measures that loaded together based on the factor analysis results and were constructed on the same scale (“Even if up” and “Regardless change”). We again analyze and report results for the composite scale and the mean as well as for each of the four measures separately.

### Experimental design

In addition to measuring current levels of support and price sensitivity with regards to CCAs, we also use a between-subjects experiment to analyze how support and price sensitivity are influenced when we show respondents educational outreach information about CCAs and energy mixes. Respondents were randomized into one of two conditions in a between-subjects design: a control group which saw no information prior to answering the outcome measures and a treatment group which saw educational outreach information about CCAs and energy mixes prior to answering the outcome measures. The treatment information included three short paragraphs describing CCAs, a question asking respondents about those three paragraphs, two short paragraphs describing energy mixes, and a question asking respondents about those two paragraphs. The questions were meant to reinforce the information the respondents had just read, in addition to serving as treatment checks.

The treatment information was constructed by condensing and simplifying information from sources such as the U.S. Environmental Protection Agency [2], the New York State Energy Research and Development Authority [35], LEAN Energy US [36], and Solstice [37]. A common strategy among organizations seeking to expand CCAs is to “raise awareness about the benefits and functional mechanics of CCA” [24]. To determine the efficacy of such informational outreach endeavors, we wanted to test if the general population could shift their attitudes in response to somewhat technical information about CCAs and energy mixes.

By adapting real materials for our treatment, we evaluate existing outreach and awareness campaigns by nonprofit and government organizations seeking to expand CCAs. Since the nature of “opt-out” programs often means that the majority of participants are included without meaningful prior knowledge of the program, we wanted to test whether showing respondents information about CCAs would influence their support and/or price sensitivity. In other words, would people still want to participate in the CCA if they knew what it was? Or are CCAs expanding dramatically despite a lack of support for CCAs? Conversely and most importantly, can informing people about CCAs increase support for CCAs? By modeling our treatment based on educational outreach information from real sources, we increase external validity and provide evidence that existing outreach endeavors are effective at increasing support.

Among the final sample of respondents (who had already passed two attention checks), we found high levels of attentiveness for the treatment information. The median length of time respondents spent reading the three short paragraphs describing CCAs was 29 seconds; accordingly, 89% of respondents correctly responded to a treatment check, stating that CCAs allow local governments to purchase energy on behalf of their residents. We are thus confident that respondents generally received the treatment as intended.

Respondents in both the control group and the treatment group then answered the outcome measures above to elicit their support for CCAs and their price sensitivity with regard to CCAs.

### Statistical methods

Following conventional practices, we used a lasso variable selection procedure to determine the set of prognostic covariates to include in models for each dependent variable [38]. We include partisanship and a treatment indicator in all regressions, given our substantive interest in these two variables. The following variables were also included in the lasso for selection as covariates: gender, education, race, ethnicity, age, ideology, political interest, trust in scientific research and statements, income, and three measures of environmental concern. We also included pretreatment measures of the three price sensitivity statements in the lasso [39, 40]. See S1 Appendix for the full survey instrument, including exact wording for all measures and stimuli.

We use principal component factor analysis to create composite scales of our dependent variable measures. Since all three measures of CCA support loaded together on the same underlying dimension (i.e., the first principal component), we created a composite scale of these three measures. Since only three of the four measures of price sensitivity with regards to CCAs loaded together on the same underlying dimension, we created a composite scale of those three measures (“Even if up,” “Regardless change,” and “Monetary benefit”).

Statistical analyses of the between-subjects experiment results were conducted using OLS regression with robust standard errors. To facilitate comparisons across models, in addition to reporting estimated effect sizes and p-values, we also report estimated effect sizes in terms of standard deviations of the outcome variable.

## Results and discussion

### Support for CCAs

Respondents in the control condition generally held neutral opinions toward CCAs at the state, local, and personal levels (S1 Table). Such indifference is unsurprising, given that most respondents likely had not heard of CCAs before and were thus unlikely to either support or oppose them: only 615 (33.3%) respondents responded “Yes” to “Do you know what a Community Choice Aggregation (CCA) is?” (Furthermore, it is possible that a portion of those respondents were answering insincerely and did not actually know about CCAs, as acquiescence bias makes it likely that respondents may answer “Yes” instead of “No” in online surveys [41, 42].) Due to the nature of “opt-out” and automatic enrollment, indifference is unlikely to reduce levels of CCA enrollment in areas that already have CCAs. However, indifference may negatively affect the existence of CCAs in the first place, as some level of public support is likely necessary to pass CCA-enabling legislation at the state level and then implement a CCA at the local level. Thus, based on prior research finding that public support plays an important role in shaping policy [20, 21], public opinion toward CCAs likely remains important for CCA formation and expansion.

**Fig 1:**
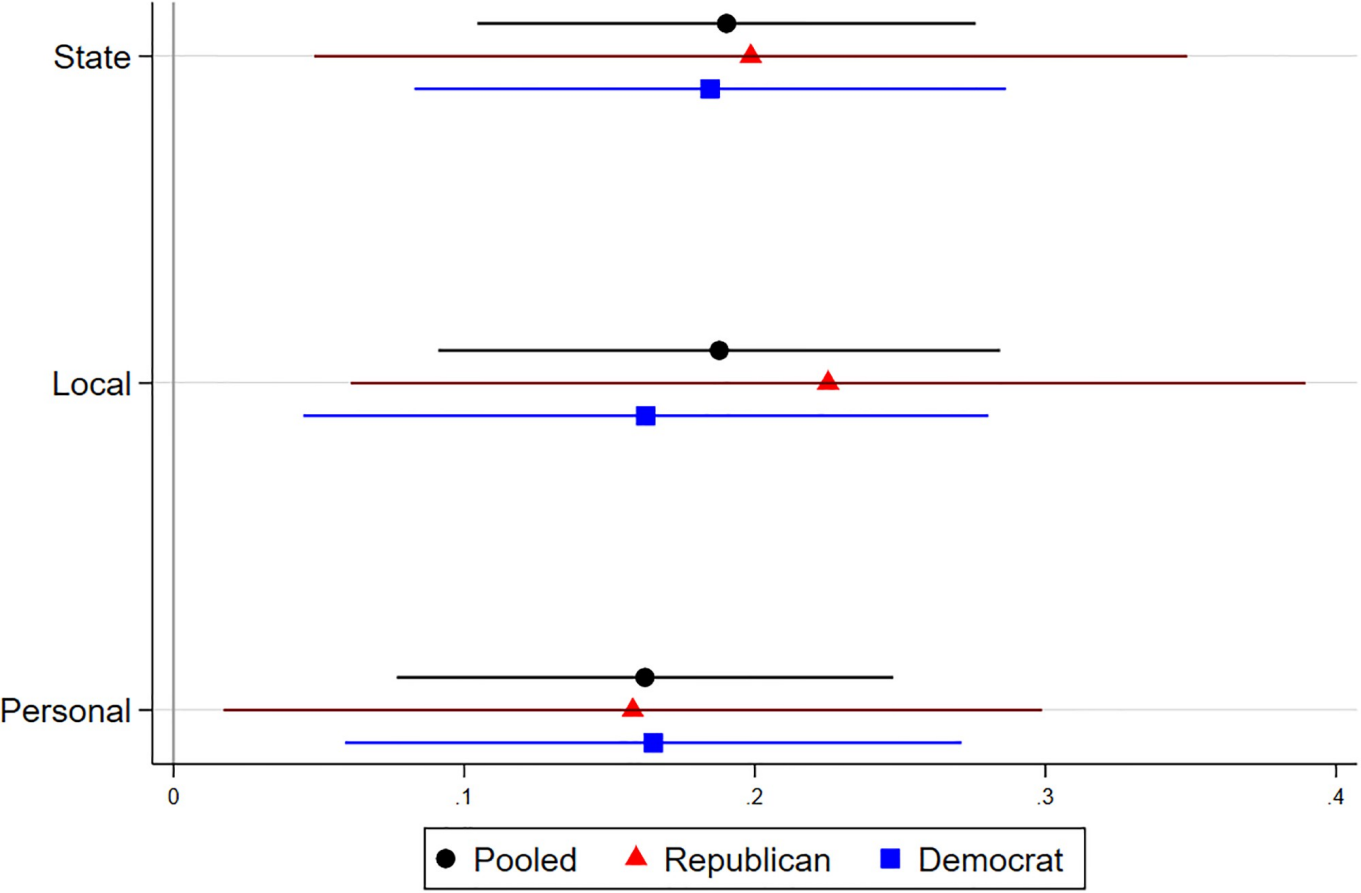
Treatment effects on support for CCAs. The horizontal bars represent the 95% confidence intervals, and estimates for which the confidence intervals do not intersect the vertical line at 0 represent statistically significant treatment effects at the 0.05 level. Pure independents are excluded; “Pooled” refers to Republicans and Democrats.

Importantly, we find that showing respondents educational outreach information about CCAs and energy mixes significantly increased their support for CCAs at the state, local, and personal levels. Table 1 and Fig 1 show that respondents in the treatment condition were significantly more likely to agree that their state should have CCA legislation (0.190, *p* <.005, 0.178 s.d.), that their local government should implement a CCA (0.188, *p* <.005, 0.151 s.d.), and that they would participate in a CCA assuming that the price of their energy would be roughly the same or slightly lower (0.162, *p* <.005, 0.156 s.d.). Treatment effects were also significant for the composite scale (0.187, *p* <.005, 0.184 s.d.) and mean (0.183, *p* <.005, 0.186 s.d.) of these three measures. In our sample, the percentage of respondents who supported CCA-enabling legislation increased from 40.2% to 53.0%, the percentage of respondents who supported their local government implementing a CCA increased from 34.4% to 47.9%, and the percentage of respondents would personally participate in a CCA increased from 49.1% to 60.6% when comparing the control and treatment groups (S2 Table).

**Table 1:** Treatment effects on support for CCAs.

	State	Local	Personal	Factor	Mean
Treatment	0.190***	0.188***	0.162***	0.187***	0.183***
	(0.044)	(0.049)	(0.044)	(0.037)	(0.036)
Republican	-0.057	-0.135*	0.036	-0.051	-0.054
	(0.052)	(0.062)	(0.054)	(0.046)	(0.045)
Controls	✓	✓	✓	✓	✓
Constant	1.218***	1.033***	1.770***	-2.250***	1.300***
	(0.188)	(0.203)	(0.191)	(0.163)	(0.160)
R^2^	0.40	0.40	0.35	0.49	0.49
N	1513	1540	1535	1531	1531

* *p* < 0.05,

** *p* < 0.01,

*** *p* <.005 (two-sided).

OLS models with robust standard errors, excluding pure independents from the sample. “State” refers to respondent support for their state adopting CCA legislation, “Local” refers to respondent support for their local government implementing a CCA, and “Personal” refers to respondents’ self-reported likelihood of participating in a CCA. “Factor” refers to a composite scale of the first three measures created using factor analysis. “Mean” refers to the mean of the first three measures. “Controls” refer to the set of prognostic covariates selected by the lasso for each dependent variable.

These results suggest that those who seek to expand renewable energy and mitigate climate change through CCA enrollment should increase existing efforts to educate the public about CCAs. Such education may increase support for CCA-enabling legislation at the state level as well as the implementation of CCAs at the local level.

We also find that treatment effects were not driven primarily by either Democrats or Republicans. Table 2 and Fig 1 show that for all measures, effects were significant for respondents of both parties. In fact, Table 2 and Fig 1 show that there was no difference in treatment effects between Democrats and Republicans (*p* >.05 for all measures)—in other words, the treatment was equally effective for Democrats and Republicans.

**Table 2:** Treatment effects on support for CCAs, with partisan interactions.

	State	Local	Personal	Factor	Mean
Treatment	0.185***	0.163**	0.165***	0.183***	0.178***
	(0.052)	(0.060)	(0.054)	(0.044)	(0.043)
Republican	-0.064	-0.167*	0.040	-0.056	-0.060
	(0.063)	(0.076)	(0.067)	(0.053)	(0.052)
Treatment × Republican	0.014	0.063	-0.007	0.010	0.012
	(0.092)	(0.103)	(0.090)	(0.078)	(0.076)
Controls	✓	✓	✓	✓	✓
Constant	1.222***	1.049***	1.769***	-2.247***	1.304***
	(0.189)	(0.203)	(0.193)	(0.164)	(0.161)
*Treatment + Treatment × Republican*					
	0.199*	0.225**	0.158*	0.193***	0.190***
	(0.077)	(0.084)	(0.072)	(0.065)	(0.063)
R^2^	0.40	0.40	0.35	0.49	0.49
N	1513	1540	1535	1531	1531

* *p* < 0.05,

** *p* < 0.01,

*** *p* <.005 (two-sided).

OLS models with robust standard errors, excluding pure independents from the sample. “State” refers to respondent support for their state adopting CCA legislation, “Local” refers to respondent support for their local government implementing a CCA, and “Personal” refers to respondents’ self-reported likelihood of participating in a CCA. “Factor” refers to a composite scale of the first three measures created using factor analysis. “Mean” refers to the mean of the first three measures. “Controls” refer to the set of prognostic covariates selected by the lasso for each dependent variable.

The tables and figures in the main text exclude pure independents from the analysis, so that the interaction term directly reflects the difference in treatment effects between Democrats and Republicans. (See the Supporting Information for tables and figures which include pure independents.) However, results remain the same when including pure independents: First, S3 Table and S1 Fig show significant treatment effects across the full sample (*p* <.005 for all five measures). Second, S4 Table and S1 Fig show that for all five measures, Republicans and Democrats increase their support for CCAs when exposed to the treatment, and there is no difference in treatment effects between Republicans and Democrats. S4 Table and S1 Fig show that there is no significant treatment effect for pure independents (*p* >.05 for all measures), but they also show that there is no difference in treatment effects between pure independents and Democrats/Republicans. Thus, the lack of significant treatment effects for pure independents on support for CCAs can likely be attributed to the relatively small number of pure independents in the final sample: the 286 pure independents made up only 15.37% of all respondents.

Finally, S3 Table includes an indicator for whether or not the respondent is from a state with CCA-enabling legislation; that covariate is significantly and positively associated with support for CCAs at the state level (*p* <.05) and the local level (*p* <.005), but not the personal level (*p* >.05). In other words, coming from a state with CCA-enabling legislation makes one more likely to support passage of CCA-enabling state legislation and implementation of a local CCA, but not one’s own participation in the CCA. It is unclear, however, which direction the causal arrow points: did states pass CCA-enabling legislation because they had a higher proportion of residents who supported CCAs? Or does living in a state with CCA-enabling legislation make one more likely to support CCAs? Furthermore, why does living in a state with CCA-enabling legislation make one more likely to support CCAs at the state and local levels but not the personal level? Future research may seek to explore these questions.

### Price sensitivity with regards to CCAs

**Fig 2:**
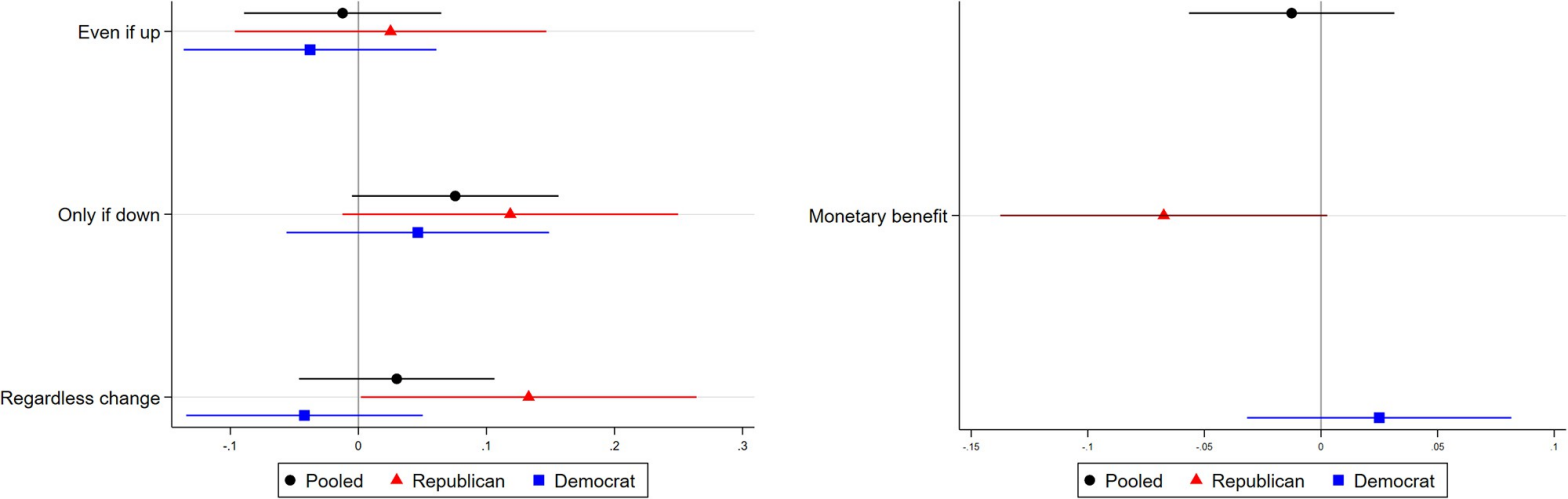
Treatment effects on price sensitivity regarding CCA participation. The horizontal bars represent the 95% confidence intervals, and estimates for which the confidence intervals do not intersect the vertical line at 0 represent statistically significant treatment effects at the 0.05 level. Pure independents are excluded; “Pooled” refers to Republicans and Democrats.

Respondents in the control condition again held generally neutral attitudes towards CCAs in terms of price sensitivity (S5 Table). However, showing respondents educational outreach information about CCAs and energy mixes did not significantly affect their price sensitivity with regard to CCAs. Table 3 and Fig 2 show that respondents in the treatment condition were not significantly more or less likely to disagree with the statement, “I would participate in a Community Choice Aggregation (CCA) **even if** my electricity bill would go up,” agree with the statement, “I would participate in a Community Choice Aggregation (CCA) **only if** my electricity bill would go down,” or disagree with the statement, “I would participate in a Community Choice Aggregation (CCA) **regardless** of how my electricity bill might change” (*p* >.05 for all three measures). (These measures are referred to as “Even if up,” “Only if down,” and “Regardless change” in tables and figures.) Respondents in the treatment condition were also not significantly more or less likely to choose “Lower electricity bill” instead of “More renewable sources of energy” or “More local control over energy” as the potential benefit of CCAs that they found most appealing (*p* >.05). (This measure is referred to as “Monetary benefit” in tables and figures. Since “Monetary benefit” was coded as a binary variable instead of on a 5-point scale, it is represented on a separate plot in figures.) Treatment effects were also null for the two aggregate measures (*p* >.05 for both).

**Table 3:** Treatment effects on price sensitivity regarding CCA participation.

	Individual measures				Aggregate measures	
	“Even if up”	“Only if down”	“Regardless change”	“Monetary benefit”	Factor	Mean
Treatment	-0.012	0.077	0.031	-0.013	-0.001	0.006
	(0.039)	(0.041)	(0.039)	(0.022)	(0.029)	(0.033)
Republican	0.000	0.017	0.017	0.032	0.037	0.030
	(0.047)	(0.055)	(0.049)	(0.028)	(0.039)	(0.045)
Controls	✓	✓	✓	✓	✓	✓
Constant	0.995***	0.443*	1.361***	0.649***	-1.140***	1.210***
	(0.182)	(0.193)	(0.193)	(0.106)	(0.167)	(0.185)
R^2^	0.67	0.44	0.62	0.26	0.70	0.71
N	1536	1510	1501	1503	1496	1521

* *p* < 0.05,

** *p* < 0.01,

*** *p* <.005 (two-sided).

OLS models with robust standard errors, excluding pure independents from the sample. Across all measures, positive coefficients imply higher price sensitivity. “Even if up” refers to how likely respondents were to disagree with the statement, “I would participate in a Community Choice Aggregation (CCA) **even if** my electricity bill would go up.” “Only if down” refers to how likely respondents were to agree with the statement, “I would participate in a Community Choice Aggregation (CCA) **only if** my electricity bill would go down.” “Regardless change” refers to how likely respondents were to disagree with the statement, “I would participate in a Community Choice Aggregation (CCA) **regardless** of how my electricity bill might change.” “Monetary benefit” refers to how likely respondents were to choose “Lower electricity bill” rather than “More renewable sources of energy” or “More local control over energy” as the potential benefit of CCAs that they found most appealing. “Factor” refers to a composite scale, created using factor analysis, of the three measures that loaded together: “Even if up,” “Regardless change,” and “Nonmonetary benefit.” “Mean” refers to the mean of the measures that loaded together and were constructed on the same scale: “Even if up” and “Regardless change.” “Controls” refer to the set of prognostic covariates selected by the lasso for each dependent variable.

With the sole exception of the “Regardless change” measure for Republicans, Table 4 and Fig 2 show that treatment effects are null for both Democrats and Republicans when considering each group separately (*p* > 0.05 for all measures except “Regardless change” for Republicans). Thus, the null treatment effects cannot be explained by the treatment affecting only Democrats or only Republicans.

**Table 4:** Treatment effects on price sensitivity regarding CCA participation, with partisan interactions.

	Individual measures				Aggregate measures	
	“Even if up”	“Only if down”	“Regardless change”	“Monetary benefit”	Factor	Mean
Treatment	-0.038	0.047	-0.040	0.025	-0.016	-0.044
	(0.050)	(0.052)	(0.047)	(0.029)	(0.036)	(0.041)
Republican	-0.032	-0.029	-0.074	0.080*	0.018	-0.040
	(0.054)	(0.063)	(0.058)	(0.036)	(0.045)	(0.050)
Treatment × Republican	0.063	0.074	0.177*	-0.092*	0.038	0.131
	(0.079)	(0.085)	(0.082)	(0.046)	(0.059)	(0.070)
Controls	✓	✓	✓	✓	✓	✓
Constant	1.012***	0.473**	1.413***	0.623***	-1.128***	1.267***
	(0.183)	(0.181)	(0.196)	(0.106)	(0.169)	(0.191)
*Treatment + Treatment × Republican*						
	0.025	0.121	0.136*	-0.067	0.022	0.087
	(0.062)	(0.067)	(0.067)	(0.036)	(0.048)	(0.056)
R^2^	0.67	0.44	0.62	0.26	0.70	0.71
N	1536	1513	1501	1503	1496	1499

* *p* < 0.05,

** *p* < 0.01,

*** *p* <.005 (two-sided).

OLS models with robust standard errors, excluding pure independents from the sample. Across all measures, positive coefficients imply higher price sensitivity. “Even if up” refers to how likely respondents were to disagree with the statement, “I would participate in a Community Choice Aggregation (CCA) **even if** my electricity bill would go up.” “Only if down” refers to how likely respondents were to agree with the statement, “I would participate in a Community Choice Aggregation (CCA) **only if** my electricity bill would go down.” “Regardless change” refers to how likely respondents were to disagree with the statement, “I would participate in a Community Choice Aggregation (CCA) **regardless** of how my electricity bill might change.” “Monetary benefit” refers to how likely respondents were to choose “Lower electricity bill” rather than “More renewable sources of energy” or “More local control over energy” as the potential benefit of CCAs that they found most appealing. “Factor” refers to a composite scale, created using factor analysis, of the three measures that loaded together: “Even if up,” “Regardless change,” and “Nonmonetary benefit.” “Mean” refers to the mean of the measures that loaded together and were constructed on the same scale: “Even if up” and “Regardless change.” “Controls” refer to the set of prognostic covariates selected by the lasso for each dependent variable.

Results remain mostly the same when including pure independents: S6 Table and S2 Fig show null treatment effects across the full sample for all six measures except for the “Only if down” measure. For the “Only if down” measure, including pure independents results in the treatment actually *increasing* price sensitivity across the full sample (0.078, *p* < 0.05). However, the lack of significant treatment effects for the other three individual measures (as well as the two aggregate measures) suggests that the treatment did not meaningfully affect respondents’ overall price sensitivity. Furthermore, treatment effects are null for Democrats, Republicans, and pure independents separately (S7 Table and S2 Fig).

Given the positive and significant treatment effect on support for CCAs, the lack of a treatment effect on price sensitivity is perhaps surprising. One might expect that the positive treatment effect on support should suggest a negative treatment effect on price sensitivity (i.e., people would care less about price after they learn about CCAs) because educational outreach information causes respondents to support the non-monetary benefits of CCAs. But we do not find evidence of this mechanism. One explanation for our combination of results is that the treatment does not increase respondents’ general support for CCAs *enough* to counteract a potential increase in their personal electricity bills. Thus, there could be a disconnect between how respondents answer when they are thinking from a societal versus a personal perspective when some cost must be incurred; the “personal” measure of CCA support did not require the respondent to incur a cost, because it specified that the price of the respondent’s energy would remain the same or be slightly lower. In other words, learning about CCAs may increase one’s support for CCAs, but not enough to reduce one’s price sensitivity with regards to CCAs.

On the other hand, price sensitivity with regards to energy costs currently favors CCAs. As previously mentioned, a structural feature of most CCAs is cost savings, as CCAs allow local governments to lower the cost of electricity by aggregating the purchasing power of their residents. CCAs do recognize the perceived importance of price—as O’Shaughnessy et al. [3] note, all the CCAs they interviewed stated that “the ability to offer electricity cost savings to customers is critical for the ongoing viability of CCAs.” CCAs thus already advertise lower prices for their default energy mix as a key advantage over IOUs. Since educational outreach information about CCAs does not affect price sensitivity one way or another, we conclude that such information can only be beneficial because they increase support for CCAs.

Finally, S6 Table includes an indicator for whether or not the respondent is from a state with CCA-enabling legislation; that covariate is not significantly associated with price sensitivity regarding CCA participation (*p* > 0.05 for all measures). In other words, people from states with and without CCA-enabling legislation do not have significantly different price sensitivities with regards to CCAs; however, as mentioned above, people from states with CCA-enabling legislation are more likely to support CCAs at the state and local levels. This set of results again begs the question: what mechanism best explains differences in support for CCAs, if not differences in price sensitivity with regards to CCAs?

## Conclusion

By adapting educational outreach information from real sources for our treatment, we evaluate the effectiveness of existing outreach and awareness campaigns by nonprofit and government organizations seeking to expand CCAs. We find that giving people educational outreach information about CCAs and energy mixes increases their support for CCAs at the state, local, and personal levels. Though giving people such information does not affect their price sensitivity with regards to CCAs, outreach information may not need to shift price sensitivity, as CCAs’ default energy mixes are generally less expensive. We further find that educational outreach information was equally effective for Republicans and Democrats. In other words, Democrats and Republicans are equally receptive to educational outreach information about an energy policy meant to increase the use of renewables—despite previous literature finding partisan polarization on renewable energy and the environment more generally. Attitudes towards CCAs have not yet become irreversibly entangled with partisan politics.

There are several limitations of this study. First, the measure of “personal” support for CCAs will inevitably underestimate how many people would participate in a CCA, due to the “opt-out” nature of CCAs. Thus, that measure represents not how many people would actually participate in a CCA in the real world, but rather respondents’ personal willingness or desire to participate in a CCA. Second, we do not test source effects. Future research might tell respondents that the message came from co-partisan or opposition partisan elites in order to test for source-based backfire effects. Finally, future research might test different types of outreach messages and analyze, for instance, whether a message focusing on cost savings might be more effective than a message focusing on renewable energy and emissions reduction.

As CCAs serve more residents and expand to more states, it becomes increasingly important to understand public opinion toward CCAs, especially in terms of partisan attitudes. Policymakers and nonprofit organizations hoping to accelerate the expansion of CCAs may modify their public outreach and/or lobbying strategies based on such research. Our results suggest that these groups should seek to increase public education and awareness about CCAs.

## Supporting information

S1 Appendix Survey instrument. (PDF)

S1 Table Support for CCAs, means and standard deviations. (PDF)

S2 Table Summary of support for CCAs. (PDF)

S3 Table Treatment effects on support for CCAs, including pure independents. (PDF)

S4 Table Treatment effects on support for CCAs, including partisan interactions and pure independents. (PDF)

S5 Table Summary of price sensitivity regarding CCAs. (PDF)

S6 Table Treatment effects on price sensitivity regarding CCA participation, including pure independents. (PDF)

S7 Table Treatment effects on price sensitivity regarding CCA participation, including partisan interactions and pure independents. (PDF)

S1 Fig Treatment effects on support for CCAs, including pure independents. The horizontal bars represent the 95% confidence intervals, and estimates for which the confidence intervals do not intersect the vertical line at 0 represent statistically significant treatment effects at the 0.05 level. “Pooled” represents pure independents, Republicans, and Democrats.
(PDF)

S2 Fig Treatment effects on price sensitivity regarding CCA participation, including pure independents. The horizontal bars represent the 95% confidence intervals, and estimates for which the confidence intervals do not intersect the vertical line at 0 represent statistically significant treatment effects at the 0.05 level. “Pooled” represents pure independents, Republicans, and Democrats.
(PDF)
