# Supplementary material for: Shifting partisan public opinion towards Community Choice Aggregation through outreach and awareness
Source: PLoS One. 2023 Oct 3;18(10):e0292136. doi: 10.1371/journal.pone.0292136 (PMC10547185; doi:10.1371/journal.pone.0292136)
Supplement: S1 Appendix — (PDF) [file pone.0292136.s001.pdf]

S1 Appendix. Survey instrument.
[Text in brackets indicates notes that the respondents did not see. Bolded/italicized text was also bolded/italicized for respondents.]

[Experimental treatment]

Please read the following text very carefully. You will be asked questions about it.

Americans in most states receive electricity from an investor-owned utility, or IOU. However, some states have Community Choice Aggregations, also known as CCAs. **CCAs are programs that allow local governments to purchase energy on behalf of their residents. Local governments with CCAs can then negotiate for lower electricity prices and/or more renewable sources of energy for residents in their communities.**

Currently, ten states have enacted CCA legislation. If a state enacts CCA legislation, then local governments in that state can decide whether they want to implement CCAs. **If a local government implements a CCA, each local resident is automatically enrolled in the CCA unless he or she decides to opt out.** Residents receive multiple notices letting them know that they can opt out of the CCA.

If a resident opts out, they will not participate in the CCA. They will continue to receive power from an investor-owned utility (IOU). But if a resident participates in the CCA, they will receive power purchased by the CCA, though the IOU will continue to maintain transmission lines and provide customer service.

[Page break]

Based on what you just read, please select the choice that correctly fills in the blank:

CCAs allow local governments to purchase ______ on behalf of their residents.

- Food
- Energy
- Housing

[Page break]

Please read the following text very carefully. You will be asked questions about it.

Electricity is usually generated by a **mix of different sources**. This mix of sources is called an "**energy mix**." Different mixes may have different prices. For example, energy mixes with more renewables are often more expensive.

A CCA's default energy mix is usually about the same price as the default mix from the IOU, or slightly lower. However, residents enrolled in a CCA have the choice to upgrade to more expensive energy mixes with a higher percentage of renewable energy and/or locally produced renewable energy.

[Page break]
Based on what you just read, please select the choice that correctly fills in the blank: The price of a CCA's default energy mix is usually ______ than the price of the default mix from the IOU.

- About the same or slightly lower
- Much higher
- Much lower

[Dependent variables]
[CCA support]
Rate your agreement with the following statement: My state should have Community Choice Aggregation (CCA) legislation, which authorizes local governments to decide whether they want to implement CCAs.

- Strongly agree
- Somewhat agree
- Neither agree nor disagree
- Somewhat disagree
- Strongly disagree

Rate your agreement with the following statement: My local government should implement a Community Choice Aggregation (CCA), which automatically enrolls each local resident in the CCA unless he or she decides to opt out.

- Strongly agree
- Somewhat agree
- Neither agree nor disagree
- Somewhat disagree
- Strongly disagree

Rate your agreement with the following statement: Assuming that the price of my energy would be roughly the same or slightly lower, I would participate in a Community Choice Aggregation (CCA).

- Strongly agree
- Somewhat agree
- Neither agree nor disagree
- Somewhat disagree
- Strongly disagree

[Price sensitivity with regard to CCAs]
Rate your agreement with the following statement: I would participate in a Community Choice Aggregation (CCA) **even if** my electricity bill would go up.

- Strongly agree
- Somewhat agree
- Neither agree nor disagree
- Somewhat disagree
- Strongly disagree

Rate your agreement with the following statement: I would participate in a Community Choice Aggregation (CCA) **only if** my electricity bill would go down.

- Strongly agree
- Somewhat agree
- Neither agree nor disagree
- Somewhat disagree
- Strongly disagree

Rate your agreement with the following statement: I would participate in a Community Choice Aggregation (CCA) **regardless** of how my electricity bill might change.

- Strongly agree
- Somewhat agree
- Neither agree nor disagree
- Somewhat disagree
- Strongly disagree

Select the potential benefit of Community Choice Aggregations (CCAs) that is most appealing to you.

- More renewable sources of energy
- Lower electricity bill
- More local control over energy

[Covariates]
[CCA state]
In which state do you currently reside?

- Alabama
- Alaska
- Arizona
- Arkansas
- California

- Colorado
- Connecticut
- Delaware
- District of Columbia
- Florida
- Georgia
- Hawaii
- Idaho
- Illinois
- Indiana
- Iowa
- Kansas
- Kentucky
- Louisiana
- Maine
- Maryland
- Massachusetts
- Michigan
- Minnesota
- Mississippi
- Missouri
- Montana
- Nebraska
- Nevada
- New Hampshire
- New Jersey
- New Mexico
- New York
- North Carolina
- North Dakota
- Ohio
- Oklahoma

- Oregon
- Pennsylvania
- Rhode Island
- South Carolina
- South Dakota
- Tennessee
- Texas
- Utah
- Vermont
- Virginia
- Washington
- West Virginia
- Wisconsin
- Wyoming
- Puerto Rico
- I do not reside in the United States

[Partisanship, ideology, and political interest]

Generally speaking, do you usually think of yourself as a Republican, a Democrat, an Independent, or something else?

- Republican
- Democrat
- Independent
- Something else

Would you call yourself a strong Republican or a not very strong Republican? [if selected Republican]

- Strong
- Not very strong

Would you call yourself a strong Democrat or a not very strong Democrat? [if selected Democrat]

- Strong

- Not very strong

Do you think of yourself as closer to the Republican or Democratic party? [if selected Independent or Something else]

- Closer to the Republican Party
- Closer to the Democratic Party
- Neither

When it comes to politics, would you describe yourself as liberal, conservative, or neither liberal nor conservative?

- Very conservative
- Somewhat conservative
- Slightly conservative
- Moderate; middle of the road
- Slightly liberal
- Somewhat liberal
- Very liberal

Generally, how interested are you in politics?

- Extremely interested
- Very interested
- Somewhat interested
- Not very interested
- Not at all interested

[Other demographics]
What is your sex?

- Male
- Female

What is the highest level of school you have completed or the highest degree you have received?

- Less than high school degree
- High school graduate (high school diploma or equivalent including GED)
- Some college but no degree
- Associate degree in college (2-year)

- Bachelor's degree in college (4-year)
- Master's degree
- Doctoral degree
- Professional degree (JD, MD)

Please check one or more categories below to indicate what race(s) you consider yourself to be.

- White
- Black or African American
- American Indian or Alaska Native
- Asian/Pacific Islander
- Multi-racial
- Other

Are you Spanish, Hispanic, or Latino or none of these?

- Spanish, Hispanic, or Latino
- None of these

How old are you?

- 18 - 24
- 25 - 34
- 35 - 44
- 45 - 54
- 55 - 64
- 65 - 74
- 75 - 84
- 85 or older

In the previous year, what was your total household income (before taxes)? If you're not sure of the specific number, please give your best guess.

- Less than $10,000
- $10,000 to $19,999
- $20,000 to $29,999
- $30,000 to $39,999
- $40,000 to $49,999

- $50,000 to $59,999
- $60,000 to $69,999
- $70,000 to $79,999
- $80,000 to $89,999
- $90,000 to $99,999
- $100,000 to $149,999
- $150,000 or more

[Environment/science covariates]

Are you very interested, moderately interested, or not at all interested in issues about environmental pollution?

- Very interested
- Moderately interested
- Not at all interested

Should federal spending on protecting the environment be increased, decreased, or kept the same?

- Increased
- Decreased
- Kept the same

Which of the following approaches would you prefer for addressing America's energy supply needs?

- Mostly developing alternative sources, such as wind, solar, and hydrogen technology
- A mix of expanding exploration and production of oil, coal, and natural gas, and developing alternative sources such as wind, solar, and hydrogen technology
- Mostly expanding exploration and production of oil, coal, and natural gas

How often would you say scientists do a good job conducting research?

- All or most of the time
- Some of the time
- Only a little of the time
- None of the time

How often would you say scientists provide fair and accurate information when communicating their research results to the public?

- All or most of the time
- Some of the time
- Only a little of the time
- None of the time

[Pre-treatment measures of price sensitivity with regard to CCAs; same as above]
Rate your agreement with the following statement: I would participate in a Community Choice Aggregation (CCA) **even if** my electricity bill would go up.

- Strongly agree
- Somewhat agree
- Neither agree nor disagree
- Somewhat disagree
- Strongly disagree

Rate your agreement with the following statement: I would participate in a Community Choice Aggregation (CCA) **only if** my electricity bill would go down.

- Strongly agree
- Somewhat agree
- Neither agree nor disagree
- Somewhat disagree
- Strongly disagree

Rate your agreement with the following statement: I would participate in a Community Choice Aggregation (CCA) **regardless** of how my electricity bill might change.

- Strongly agree
- Somewhat agree
- Neither agree nor disagree
- Somewhat disagree
- Strongly disagree

[Other variable reported in the text]

Do you know what a Community Choice Aggregation (CCA) is?

- Yes
- No
