# Supplementary material for: Shifting partisan public opinion towards Community Choice Aggregation through outreach and awareness
Source: PLoS One. 2023 Oct 3;18(10):e0292136. doi: 10.1371/journal.pone.0292136 (PMC10547185; doi:10.1371/journal.pone.0292136)
Supplement: S1 Table — (PDF) [file pone.0292136.s002.pdf]

S1 Table. Support for CCAs, means and standard deviations.

	Untreated			Treated		
	Pooled	Democrat	Republican	Pooled	Democrat	Republican
State	3.374	3.717	2.984	3.584	3.896	3.253
	(1.019)	(0.930)	(1.069)	(1.123)	(0.984)	(1.216)
Local	3.136	3.550	2.686	3.367	3.711	2.987
	(1.189)	(1.074)	(1.212)	(1.240)	(1.150)	(1.304)
Personal	3.548	3.820	3.255	3.712	3.974	3.478
	(0.965)	(0.914)	(1.006)	(1.075)	(0.979)	(1.153)
N	934	467	322	927	470	316

Standard deviations in parentheses. Respondents rated their agreement on a 5-point scale from "Strongly disagree" (1) to "Strongly agree" (5). "State" refers to agreement with the statement, "My state should have Community Choice Aggregation (CCA) legislation, which authorizes local governments to decide whether they want to implement CCAs." "Local" refers to agreement with the statement, "My local government should implement a Community Choice Aggregation (CCA), which automatically enrolls each local resident in the CCA unless he or she decides to opt out." "Personal" refers to agreement with the statement, "Assuming that the price of my energy would be roughly the same or slightly lower, I would participate in a Community Choice Aggregation (CCA)." "Pooled" represents pure independents, Republicans, and Democrats.
