# Supplementary material for: Shifting partisan public opinion towards Community Choice Aggregation through outreach and awareness
Source: PLoS One. 2023 Oct 3;18(10):e0292136. doi: 10.1371/journal.pone.0292136 (PMC10547185; doi:10.1371/journal.pone.0292136)
Supplement: S2 Table — (PDF) [file pone.0292136.s003.pdf]

S2 Table. Summary of support for CCAs.

	State		Local		Personal	
	Untreated	Treated	Untreated	Treated	Untreated	Treated
Strongly disagree	55	57	115	96	36	50
	(5.9%)	(6.1%)	(12.3%)	(10.4%)	(3.9%)	(5.4%)
Somewhat disagree	63	68	112	114	43	46
	(6.7%)	(7.3%)	(12.0%)	(12.3%)	(4.6%)	(5.0%)
Neither agree nor disagree	441	311	386	273	396	269
	(47.2%)	(33.5%)	(41.3%)	(29.4%)	(42.4%)	(29.0%)
Somewhat agree	228	259	173	242	291	318
	(24.4%)	(27.9%)	(18.5%)	(26.1%)	(31.2%)	(34.3%)
Strongly agree	147	232	148	202	168	244
	(15.7%)	(25.0%)	(15.8%)	(21.8%)	(18.0%)	(26.3%)
Total	934	927	934	927	934	927

"State" refers to the statement, "My state should have Community Choice Aggregation (CCA) legislation, which authorizes local governments to decide whether they want to implement CCAs." "Local" refers to the statement, "My local government should implement a Community Choice Aggregation (CCA), which automatically enrolls each local resident in the CCA unless he or she decides to opt out." "Personal" refers to the statement, "Assuming that the price of my energy would be roughly the same or slightly lower, I would participate in a Community Choice Aggregation (CCA)."
