# Supplementary material for: Shifting partisan public opinion towards Community Choice Aggregation through outreach and awareness
Source: PLoS One. 2023 Oct 3;18(10):e0292136. doi: 10.1371/journal.pone.0292136 (PMC10547185; doi:10.1371/journal.pone.0292136)
Supplement: S3 Table — (PDF) [file pone.0292136.s004.pdf]

**S3 Table. Treatment effects on support for CCAs, including pure independents.**

| | State | Local | Personal | Factor | Mean |
|---|---|---|---|---|---|
| Treatment | 0.176*** | 0.170*** | 0.133*** | 0.167*** | 0.161*** |
| | (0.040) | (0.046) | (0.040) | (0.035) | (0.034) |
| Republican | -0.004 | -0.034 | 0.110 | 0.035 | 0.032 |
| | (0.064) | (0.078) | (0.064) | (0.057) | (0.055) |
| Democrat | 0.080 | 0.157* | 0.074 | 0.103 | 0.101 |
| | (0.062) | (0.073) | (0.061) | (0.055) | (0.053) |
| CCA state | 0.103* | 0.194*** | 0.063 | 0.122*** | 0.121*** |
| | (0.043) | (0.052) | (0.043) | (0.038) | (0.036) |
| Controls | ✓ | ✓ | ✓ | ✓ | ✓ |
| Constant | 2.349*** | 2.499*** | 1.821*** | -1.255*** | 2.262*** |
| | (0.210) | (0.259) | (0.205) | (0.182) | (0.171) |
| $R^2$ | 0.38 | 0.39 | 0.33 | 0.47 | 0.47 |
| N | 1806 | 1766 | 1778 | 1806 | 1806 |

* $p < 0.05$, ** $p < 0.01$, *** $p < .005$ (two-sided). OLS models with robust standard errors, including pure independents in the sample. The "CCA state" covariate refers to whether or not the respondent is from a state that has passed CCA-enabling legislation. "State" refers to respondent support for their state adopting CCA legislation, "Local" refers to respondent support for their local government implementing a CCA, and "Personal" refers to respondents' self-reported likelihood of participating in a CCA. "Factor" refers to a composite scale of the first three measures created using factor analysis. "Mean" refers to the mean of the first three measures. "Controls" refer to the set of prognostic covariates selected by the lasso for each dependent variable.
