# Supplementary material for: Shifting partisan public opinion towards Community Choice Aggregation through outreach and awareness
Source: PLoS One. 2023 Oct 3;18(10):e0292136. doi: 10.1371/journal.pone.0292136 (PMC10547185; doi:10.1371/journal.pone.0292136)
Supplement: S5 Table — (PDF) [file pone.0292136.s006.pdf]

S5 Table. Summary of price sensitivity regarding CCAs.

	"Even if up"		"Only if down"		"Regardless change"		"Monetary benefit"	
	Untreated	Treated	Untreated	Treated	Untreated	Treated	Untreated	Treated
Strongly disagree	194 (20.8%)	179 (19.3%)	40 (4.3%)	43 (4.6%)	118 (12.6%)	125 (13.5%)		
Somewhat disagree	153 (16.4%)	195 (21.0%)	62 (6.6%)	69 (7.4%)	120 (12.9%)	145 (15.6%)		
Neither agree nor disagree	334 (35.8%)	246 (26.5%)	328 (35.2%)	238 (25.6%)	427 (45.8%)	350 (37.7%)		
Somewhat agree	131 (14.0%)	177 (19.1%)	297 (31.8%)	335 (36.1%)	132 (14.1%)	180 (19.4%)		
Strongly agree	122 (13.1%)	131 (14.1%)	206 (22.1%)	243 (26.2%)	136 (14.6%)	128 (13.8%)		
Lower electricity bill							495 (53.2%)	484 (52.2%)
More local control over energy							105 (11.3%)	92 (9.9%)
More renewable sources of energy							331 (35.6%)	351 (37.9%)
Total	934	928	933	928	933	928	931	927

"Even if up" refers to the statement, "I would participate in a Community Choice Aggregation (CCA) **even if** my electricity bill would go up." "Only if down" refers to the statement, "I would participate in a Community Choice Aggregation (CCA) **only if** my electricity bill would go down." "Regardless change" refers to the statement, "I would participate in a Community Choice Aggregation (CCA) **regardless** of how my electricity bill might change." "Monetary benefit" refers to which potential benefit of CCAs that respondents found most appealing.
