# Supplementary material for: Shifting partisan public opinion towards Community Choice Aggregation through outreach and awareness
Source: PLoS One. 2023 Oct 3;18(10):e0292136. doi: 10.1371/journal.pone.0292136 (PMC10547185; doi:10.1371/journal.pone.0292136)
Supplement: S6 Table — (PDF) [file pone.0292136.s007.pdf]

S6 Table. Treatment effects on price sensitivity regarding CCA participation, including pure independents.

	Individual measures				Aggregate measures	
	"Even if up"	"Only if down"	"Regardless change"	"Monetary benefit"	Factor	Mean
Treatment	0.004	0.078*	0.044	-0.007	0.013	0.024
	(0.036)	(0.038)	(0.036)	(0.021)	(0.027)	(0.031)
Republican	-0.009	0.036	-0.049	0.033	-0.002	-0.029
	(0.057)	(0.062)	(0.058)	(0.036)	(0.044)	(0.049)
Democrat	-0.034	0.030	-0.083	-0.003	-0.038	-0.054
	(0.054)	(0.059)	(0.051)	(0.035)	(0.042)	(0.045)
CCA state	-0.047	0.063	0.006	0.036	0.009	-0.021
	(0.038)	(0.041)	(0.038)	(0.022)	(0.029)	(0.033)
Controls	✓	✓	✓	✓	✓	✓
Constant	1.093***	0.431*	1.414***	0.596***	-1.228***	1.237***
	(0.171)	(0.173)	(0.193)	(0.094)	(0.146)	(0.165)
R^2	0.66	0.44	0.61	0.24	0.69	0.70
N	1800	1780	1766	1771	1762	1764

* $p < 0.05$, ** $p < 0.01$, *** $p < .005$ (two-sided). OLS models with robust standard errors, including pure independents in the sample. Across all measures, positive coefficients imply higher price sensitivity. The "CCA state" covariate refers to whether or not the respondent is from a state that has passed CCA-enabling legislation. "Even if up" refers to how likely respondents were to disagree with the statement, "I would participate in a Community Choice Aggregation (CCA) **even if** my electricity bill would go up." "Only if down" refers to how likely respondents were to agree with the statement, "I would participate in a Community Choice Aggregation (CCA) **only if** my electricity bill would go down." "Regardless change" refers to how likely respondents were to disagree with the statement, "I would participate in a Community Choice Aggregation (CCA) **regardless** of how my electricity bill might change." "Monetary benefit" refers to how likely respondents were to choose "Lower electricity bill" rather than "More renewable sources of energy" or "More local control over energy" as the potential benefit of CCAs that they found most appealing. "Factor" refers to a composite scale, created using factor analysis, of the three measures that loaded together: "Even if up," "Regardless change," and "Nonmonetary benefit." "Mean" refers to the mean of the measures that loaded together and were constructed on the same scale: "Even if up" and "Regardless change." "Controls" refer to the set of prognostic covariates selected by the lasso for each dependent variable.
