# Supplementary material for: Shifting partisan public opinion towards Community Choice Aggregation through outreach and awareness
Source: PLoS One. 2023 Oct 3;18(10):e0292136. doi: 10.1371/journal.pone.0292136 (PMC10547185; doi:10.1371/journal.pone.0292136)

**S1 Fig. Treatment effects on support for CCAs, including pure independents.** The horizontal bars represent the 95% confidence intervals, and estimates for which the confidence intervals do not intersect the vertical line at 0 represent statistically significant treatment effects at the 0.05 level. “Pooled” represents pure independents, Republicans, and Democrats.

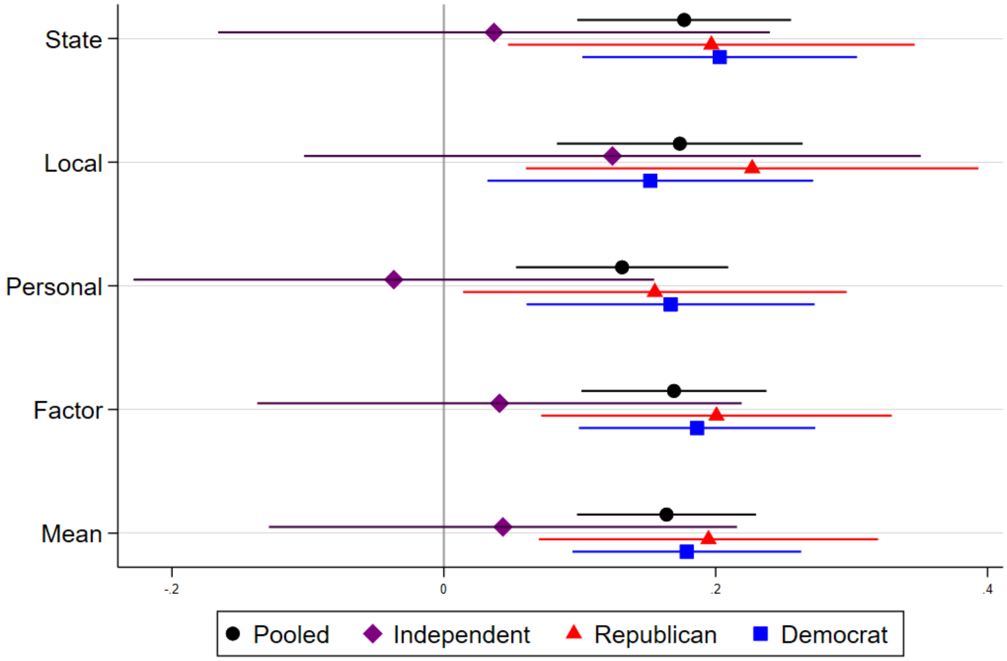

Supplement: S1 Fig — The horizontal bars represent the 95% confidence intervals, and estimates for which the confidence intervals do not intersect the vertical line at 0 represent statistically significant treatment effects at the 0.05 level. “Pooled” represents pure independents, Republicans, and Democrats. (PDF) [file pone.0292136.s009.pdf]
